# Stauffer Syndrome: Another Strange Case of the Internist's Tumor

**DOI:** 10.7759/cureus.42307

**Published:** 2023-07-22

**Authors:** Felipe A Muñoz Rossi, Diana Gallo, Ana Maria Guaiquil, Ralf Suarez, Gina Paola Ricardo Ossio

**Affiliations:** 1 Internal Medicine, National University of Colombia, Bogota, COL; 2 Medical Affairs, Universidad Militar Nueva Granada, Bogota, COL; 3 Research in Health Sciences, Universitat Internacional de Catalunya, Catalunya, ESP; 4 Medical Affairs, Universidad Metropolitana de Barranquilla, Medellin, COL; 5 Medical Affairs, Universidad del Rosario, Bogota, COL

**Keywords:** renal cell carcinoma, jaundice, cholestasis, paraneoplastic syndromes, stauffer's syndrome

## Abstract

Stauffer syndrome is a paraneoplastic disorder associated with renal cell carcinoma (RCC), which often can be the first manifestation of the tumor. It is characterized by nephrogenic hepatic dysfunction that reverses after removing the primary tumor. Stauffer syndrome has been associated with a worse prognosis in patients with RCC. We are presenting a case of a patient with an uncommon cholestatic jaundice variant. The patient also had hepatic profile alterations, but no metastasis existed. These findings meet the diagnostic criteria for Stauffer syndrome in the context of RCC as the first clinical manifestation in the diagnostic approach to malignancy-associated disease. A 74-year-old patient, with a history of obesity, hypertension, and type 2 diabetes mellitus, went for advice due to right upper abdominal pain, icterus accompanied by emetic episodes, and a cholestatic pattern in the hepatic profile. Autoimmune hepatitis was ruled out based on immunological testing. Imaging revealed evidence of a mass in the lower pole of the right kidney, suspicious of malignant neoplasia, and a distant paraneoplastic syndrome consistent with the cholestatic variant of Stauffer syndrome. This is an exciting case of Stauffer syndrome as the initial presentation of RCC associated with a cholestatic variant, providing relevant information about this uncommon condition in our setting.

## Introduction

Stauffer syndrome is a paraneoplastic disorder associated with renal cell carcinoma (RCC). It is characterized by hepatic dysfunction in the absence of metastasis or direct tumor compression, as well as alterations in hepatic profile, such as hypoalbuminemia, hypergammaglobulinemia, elevated transaminases, high alkaline phosphatase levels, and prolonged thrombin time with normalization of liver function tests after primary tumor resection [1].

Stauffer syndrome is considered unusual; however, there are no available data on its incidence [1]. The literature estimates it to be around 15% in case series [2]. In another study, approximately 7% reported non-metastatic hepatic dysfunction [3]. Stauffer syndrome was significantly associated with a worse prognosis in RCC patients [4].

The aim of this article is to present the rare case of Stauffer syndrome characterized by an alteration in liver profile, specifically cholestatic hyperbilirubinemia. It is important to highlight that this case is exceptional due to the presence of jaundice in the absence of liver metastasis in a patient with a renal mass in the lower pole of the right kidney. The patient was treated with surgical resection of this malignant neoplasm, which has been reported as RCC.

## Case presentation

A 74-year-old female, with a medical history of hypertension, obesity, and type 2 diabetes mellitus, presented to the emergency department with a three-hour history of right hypochondrium abdominal pain. The pain had a progressive onset, accompanied by nausea and food content vomiting, without other associated symptoms. Physical examination revealed jaundice; the abdomen was also tender to superficial and deep palpation of the right hypochondrium. Murphy’s sign was negative.

Laboratory tests recorded in Table 1 were performed. The results revealed high bilirubin levels, with an obstructive pattern and high alkaline phosphatase. Tests for hepatotropic viruses were negative, and the ferritin levels were elevated with a negative ferrokinetic study for hemochromatosis. The patient also presented with acute kidney injury classified as KDIGO stage 3, classified as prerenal due to the BUN-to-creatinine ratio. The autoimmune profile showed negative results for antinuclear antibodies, anti-mitochondrial antibodies, and anti-smooth muscle antibodies.

**Table 1:** Laboratory tests.

Laboratory tests	
Alanine aminotransferase (ALT)	184.0 U/L (reference values: 0-31)
Aspartate aminotransferase (AST)	46.2 U/L (reference values: 0-32)
Alkaline phosphatase	350.20 U/L (reference values: 35-104)
Total bilirubin	3.09 mg/dl (reference values: 0-1.0)
Direct bilirubin	2.98 mg/dl
Indirect bilirubin	0.11 mg/dl
Transferrin saturation	16 %
Iron-binding capacity	251 ug/dl (reference values: 228-428)
Albumin	2.94 g/dl
Gamma-glutamyl transferase	55 mm/hr
Ceruloplasmin	108.27 U/l
Antinuclear antibodies	35.56 mg/dl (reference values: 20-60)
Anti-mitochondrial antibodies	Negative
Anti-smooth muscle antibodies	Negative
Alanine aminotransferase (ALT)	Negative

According to the abdominal magnetic resonance cholangiopancreatography (MRCP), there is a hypervascular focal lesion in the lower pole of the right kidney that strongly indicates RCC. However, there are no indications of acute cholecystitis or choledocholithiasis. 

**Figure 1:**
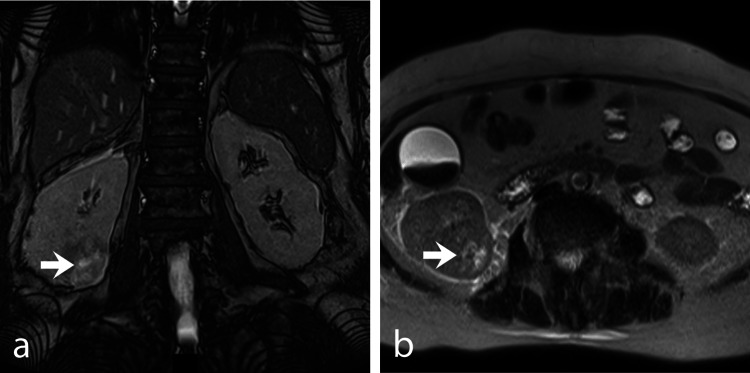
Contrast-enhanced abdominal magnetic resonance imaging. a) Mass in the lower pole of the right kidney, coronal section. b) Axial section showing the same mass in the lower pole of the right kidney.

**Figure 2:**
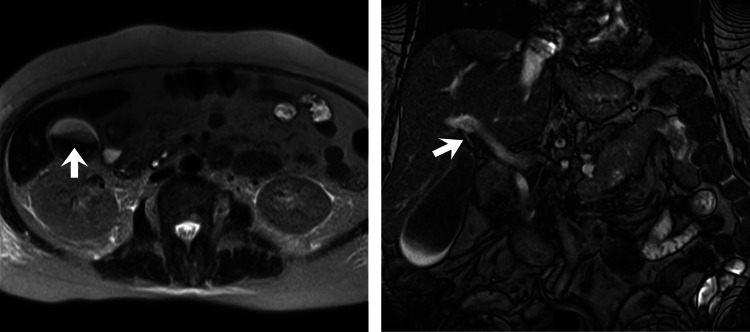
Contrast-enhanced abdominal magnetic resonance imaging. Gallbladder sludge: represented by an arrowhead in the image. Mild dilatation of the extrahepatic bile duct: indicated by a white arrow in the image.

The contrast-enhanced abdominal magnetic resonance imaging reported the presence of a right renal mass, suggestive of neoplastic involvement (Figure 1). Biliary sludge and mild dilation of the extrahepatic bile duct were also observed (Figure 2).

The Uro-CT scan confirmed the presence of a complex mass or cyst in the lower pole of the right kidney, with a dense image suggestive of biliary sludge in the gallbladder, with minimal perivascular inflammatory changes.

A biopsy of the right kidney was performed, revealing RCC with a Fuhrman/WHO-ISUP grade 2. The biopsy indicated the involvement of the renal capsule without exceeding it and not extending to the perirenal fat or renal sinus or lymphovascular invasion. The ureter, renal artery, and renal vein margins were tumor involvement-free, and non-tumoral kidneys have focal interstitial nephritis and glomerular sclerosis.

The initial clinical presentation was consistent with biliary colic. However, further evaluation revealed hepatitis with a cholestatic pattern. Serology tests for hepatotropic viruses were negative. Therefore, the differential diagnosis included cholecystitis, choledocholithiasis, and autoimmune hepatitis. The diagnosis was ruled out based on a magnetic resonance cholangiopancreatography and an adverse autoimmune profile. The urology and internal medicine service evaluated the patient, who considered the diagnosis highly suggestive of malignant neoplasm. The patient underwent surgical resection, specifically a total nephrectomy of the right kidney, which confirmed the pathological diagnosis of RCC. After the operation, the transaminitis and cholestatic symptoms resolved.

## Discussion

Stauffer syndrome, also known as nephrogenic hepatic dysfunction syndrome, is a rare condition that can be a sign of RCC. It usually presents as a form of liver disease called anicteric cholestasis [5]. Its association with paraneoplastic cholestatic jaundice is extremely rare, presenting as a variant of Stauffer syndrome (Table 2), with few cases reported in the current medical literature [6]. 

**Table 2:** Characteristics of Stauffer syndrome and its cholestatic variant. Fontes-Sousa et al. [1] (Urol Oncol Semin Orig Investig. julio de 2018;36:321-6)

Clinical/analytical	Classic description of Stauffer syndrome	Description of the S. Stauffer cholestatic variant
Alkaline phosphatase	High	High
y-Glutamyl transferase	High	High
Albumin	Low	Low
Erythrocyte sedimentation rate	High	High
a-2-globulin	High	High
Platelets	High	High (it can be associated with thrombocytopenia)
Prolonged prothrombin time	Present	Present
Hepatosplenomegaly	Present	Present
Hyperbilirubinemia	Absent	Present
Jaundice	Absent	Absent/Present
Urinary hyperpigmentation	Absent	Present
Pruritus	Absent	Present
Resolution after tumor resection	Yes	Yes

The Stauffer syndrome, first discovered by gastroenterologist Maurice H. Stauffer in 1961, has been linked to the identification of RCC, previously known as hypernephroma [7], now known as RCC. Over the years, there have been indications of a potential correlation between hypernephroma and non-metastatic liver disease [8].

The physiopathology of the syndrome remains uncertain. There is a clear connection between elevated levels of interleukin 6 (IL-6) and the development of tumors, such as cholestasis, in patients with RCC [9]. This connection has also been observed in other types of cancer, including bronchogenic carcinomas, prostate cancer, soft tissue sarcomas, pancreatic cancer, and lymphoproliferative diseases. However, the exact nature of this relationship is not as well-established in these other types of cancer [10-14].

There exists a theory that explains the mechanisms behind hepatic dysfunction. It suggests the release of a humoral substance or lysosomal enzymes from the tumor, with distant effects on the liver and hematopoietic system. The increased activity of lysosomal enzymes in the hepatic cells of patients with renal carcinoma supports this theory [15].

The patient in question presented with a rare form of cholestatic jaundice and displayed abnormalities in their liver function tests. However, there were no indications of cancer metastasis. The combination of these symptoms met the criteria for Stauffer syndrome in the context of RCC [1,8,16]. It serves as an initial clinical approach for identifying associated malignancies. 

The diagnostic algorithms utilized were comparable to those recommended by Elseidy et al. in 2022 [17]. The tests confirmed liver dysfunction with a cholestatic pattern linked to jaundice. Exclusion criteria mainly included obstructive biliary tract disease, alcoholism, autoimmune disease, and viral hepatitis. In addition, the diagnosis was verified through imaging, specifically MRCP, which detected a renal mass suspected to be malignant despite the absence of obstructive biliary disease. The presence of hypergammaglobulinemia and hypoalbuminemia supported the diagnosis of Stauffer syndrome.

An increase in alkaline phosphatase was detected, being the most frequent laboratory discovery found in 90% of cases. Elevated transaminases and hyperbilirubinemia are observed in 21% and 15% of cases, respectively [18].

In this case, the patient presented with a neoplastic growth located in the lower pole of the right kidney, which is a common presentation among many recorded cases, showing a primary renal tumor on the right side. This could suggest that anatomical proximity is a potential causal factor. However, cases are also reported on the left side [16,19]. After 30 days, the patient underwent a right nephrectomy, which confirmed the diagnosis of clear RCC. The patient experienced a complete resolution of symptoms following the intervention.

Among the limitations of this case, the early presentation of symptoms and jaundice in the patient should be noted as necessary information; however, we do not have previous laboratory test results, including the patient's biochemical profile. Furthermore, there was no subsequent follow-up to assess the patient's long-term prognosis. Differentiating this syndrome from jaundice caused by hepatic metastatic infiltration is crucial since the latter has a significantly worse prognosis [15].

## Conclusions

In conclusion, this case highlights the importance of Stauffer syndrome as the initial manifestation of RCC, previously referred to as the "internist's tumor." Diagnosing it can be challenging due to its varied and non-specific symptoms that may occur at a distance from the affected area.

Furthermore, the diagnostic confirmation through pathological examination of the nephrectomy is worth noting to confirm RCC. Therefore, based on the imaging evidence, prognosis, and disease progression, this is a unique case of a cholestatic variant of Stauffer syndrome without evident hepatic metastases, which should be considered among the top differential diagnoses.
