# Correction: Stauffer Syndrome: Another Strange Case of the Internist's Tumor

**DOI:** 10.7759/cureus.c189

**Published:** 2024-08-14

**Authors:** Felipe A Muñoz Rossi, Diana Gallo, Ana Maria Guaiquil, Ralf Suarez, Gina Paola Ricardo Ossio

**Affiliations:** 1 Internal Medicine, National University of Colombia, Bogota, COL; 2 Medical Affairs, Universidad Militar Nueva Granada, Bogota, COL; 3 Research in Health Sciences, Universitat Internacional de Catalunya, Catalunya, ESP; 4 Medical Affairs, Universidad Metropolitana de Barranquilla, Medellin, COL; 5 Medical Affairs, Universidad del Rosario, Bogota, COL

This article has been corrected to state that Stauffer syndrome was first discovered by Dr. Maurice H. Stauffer. The original published version misidentified him as Dr. Herbert Maurice Stauffer, who has no relation to Stauffer Syndrome. The authors and journal regret that this error was not identified prior to publication.

